# Consanguineous Marriages

**Published:** 1867-08

**Authors:** 


CONSANGUINEOUS MARRIAGES.—We would direct the attention of our readers to the annexed circular of Dr. R. Newman, one of a committee appointed by the New York State Medical Society, "to investigate and report upon the results of consanguineous marriages." This subject has, from time past, elicited the deepest interest throughout the medical profession, and its importance demands from every physician a communication of such facts as may have come under his observation, thus promoting a final decision of this question.

118 W. HOUSTON STREET,
NEW YORK, July, 1867.

SIR—At the late meeting of the "Medical Society of the State of New York," it was resolved: "That a Committee be appointed to investigate and report upon the result of consanguineous marriages, etc." If such marriages come under your observation, you will confer a favor by answering the following questions, and transmitting such report, before November next, to the undersigned, one of the Committee appointed:

1. Name (initials) and age of husband; 2. Nativity; 3. Age when married; 4. Constitution; 5. Health, deformities, peculiar diathesis; 6. Health of his family, hereditary diseases, deformities, etc.; 7. Name (initials) and age of wife; 8. Nativity; 9. Age when married; 10. Constitution; 11. Health, deformities, peculiar diathesis; 12. Health of her family, hereditary diseases, deformities, etc.; 13. How are the parties related to each other? 14. How long married? 15. How many children, or sterility? 16. Abortions, cause, how many, and at what period? 17. Children died, at what ages, and from what disease? 18. The constitution, age and present health of living children, deformities, mental conditions, idiocy, cretinism, deaf, mute, blind, epilepsy, albinism, insane, etc.; 19. Remarks and other information.

Hoping to receive your valuable coöperation for the advancement of medical science,

I remain yours, most respecfully,

ROBERT NEWMAN, M.D.

